# Non-Exosomal and Exosome-Derived miRNAs as Promising Biomarkers in Canine Mammary Cancer

**DOI:** 10.3390/life12040524

**Published:** 2022-04-01

**Authors:** Patrícia Petroušková, Nikola Hudáková, Marcela Maloveská, Filip Humeník, Dasa Cizkova

**Affiliations:** 1 Centre of Experimental and Clinical Regenerative Medicine, The University of Veterinary Medicine and Pharmacy, Komenského 73, 041 81 Košice, Slovakia; patricia.petrouskova@uvlf.sk (P.P.); nikola.hudakova@student.uvlf.sk (N.H.); marcela.maloveska@uvlf.sk (M.M.); filip.humenik@uvlf.sk (F.H.); 2 Institute of Neuroimmunology, Slovak Academy of Sciences, Dúbravská Cesta 9, 845 10 Bratislava, Slovakia

**Keywords:** canine, mammary cancer, biomarker, miRNA, exosome

## Abstract

Canine mammary cancer (CMC), similar to human breast cancer (HBC) in many aspects, is the most common neoplasm associated with significant mortality in female dogs. Due to the limited therapy options, biomarkers are highly desirable for early clinical diagnosis or cancer progression monitoring. Since the discovery of microRNAs (miRNAs or miRs) as post-transcriptional gene regulators, they have become attractive biomarkers in oncological research. Except for intracellular miRNAs and cell-free miRNAs, exosome-derived miRNAs (exomiRs) have drawn much attention in recent years as biomarkers for cancer detection. Analysis of exosomes represents a non-invasive, pain-free, time- and money-saving alternative to conventional tissue biopsy. The purpose of this review is to provide a summary of miRNAs that come from non-exosomal sources (canine mammary tumor, mammary tumor cell lines or canine blood serum) and from exosomes as promising biomarkers of CMC based on the current literature. As is discussed, some of the miRNAs postulated as diagnostic or prognostic biomarkers in CMC were also altered in HBC (such as miR-21, miR-29b, miR-141, miR-429, miR-200c, miR-497, miR-210, miR-96, miR-18a, miR19b, miR-20b, miR-93, miR-101, miR-105a, miR-130a, miR-200c, miR-340, miR-486), which may be considered as potential disease-specific biomarkers in both CMC and HBC.

## 1. Canine Mammary Cancer

### 1.1. General Information and Risk Factors

In veterinary medicine, canine cancer represents a severe clinical problem [1]. Approximately one in four dogs suffers cancer at some stage in their life and almost half of dogs over the age of 10 die due to neoplasia [2]. Tumors of the mammary glands are the second most commonly diagnosed type of cancer in dogs, with certain breeds, such as Labrador Retrievers, Cocker Spaniels, Irish Setters, German Shepherds, mixed-breed dogs, and miniature and Toy Poodles, and are over-represented in terms of high risk of neoplasia and mortality [3]. Mammary tumors typically develop in intact female dogs or elderly spayed (ovariohysterectomised) bitches, usually between 8 and 10 years old [4,5]. This problem is especially significant in Europe, where bitches are usually spayed at an older age [4]. However, the precise prevalence of canine mammary tumors differs from study to study due to the dog breeds and age and geographic location [6]. Biaoni et al. reported that canine mammary tumors were the most frequent tumor in Italy, wherein 476 cases per 100,000 dog-years at risk were malignant [7]. On the other hand, the incidence of mammary tumors in Sweden is higher and ranges from 111 to 154 per 10,000 dog-years at risk [8].

The incidence of canine mammary gland tumors is related to two main risk factors: age and time of exposure to ovarian hormones [9]. A study performed by Egenvall et al. describes that in bitches at ages 6, 8, and 10 years, the incidence of mammary cancer increased from 1% to 6% and 13%, respectively [8]. Ovarian hormones (estrogen, progesterone) may lead to carcinogenesis and mammary hyperplasia. Thus, ovariohysterectomy is precautionary for tumor development, and its timing seems to be crucial [10,11]. A female dog of any breed castrated before the first ovarian cycle has a 0.5% chance of developing a tumor. If the bitch is spayed just after or at any subsequent ovarian cycle, the risk of mammary gland cancer increases from 8% to 26% [9,10]. Additionally, the study of Schneider et al. demonstrated that ovariohysterectomy of bitches after their second estrum had no preventive impact against the development of malignant tumors [10].

Canine studies also indicated that obesity is another major risk factor for mammary tumor development, especially if present early in a dog’s life [12,13]. The study of Sonnenschein et al. demonstrated that a thin physique reduced the risk of mammary cancer among spayed dogs by 99%, and non-spayed dogs by 40% [12]. The influence of the diet was also studied. Dogs on a homemade diet with high-red meat portions were at a higher risk of developing mammary dysplasia and tumors compared to a commercial diet [13]. Therefore, nutritional factors, operating especially early in life, also have etiological importance to canine mammary cancer (CMC) development.

### 1.2. Classification System

Mammary glands are a frequent location for tumor development and, as in other types of cancer, canine mammary tumors may be benign or malignant [14]. Since approximately half of mammary tumors in dogs are malignant with a high percentage of mortality if not treated in time, there is no doubt that canine mammary neoplasia represents a serious clinical issue [4,15,16]. Histopathology and biopsy remain the cornerstone and the gold standards for the diagnosis and classification of canine mammary tumors [17]. However, the morphological heterogeneity of these tumors, with frequent presence of various cell populations, is challenging when providing an appropriate classification [17]. Nowadays, veterinary pathologists have available two systems of a histological classification scheme for canine mammary tumors: the official histological classification approved by the World Health Organization (WHO) and the Armed Forces Institute of Pathology from 1999 [18] and the international consensus histological classification scheme based on 2011 updates to the WHO HBC parameters proposed by Goldschmidt et al. from 2011 [19]. The latest 2011 system combines various criteria for subtyping mammary tumors by separating benign forms from malignant lesions and determining the tissue of origin (epithelial, myoepithelial, mesenchymal). A comparison of the two classification systems by Canadas et al. demonstrated that the WHO and 2011 classification systems were very similar in terms of the categorization of benign tumors, and both were prognostically relevant by identifying malignant tumors [17]. Therefore, veterinary pathologists should include both classification systems in the diagnosis and classification of canine mammary tumors.

Based on the tissue of origin, mammary gland tumors of purely epithelial origin are malignant carcinomas, such as carcinoma in situ, simple carcinoma (tubular, tubulopapillary, cystic-papillary, cybriform), solid carcinoma, anaplastic carcinoma, ductal carcinoma, complex and mixed type carcinoma [17,18]. However, there are also other special types of malignant epithelial neoplasms (squamous cell carcinoma, adenosquamous carcinoma, mucinous carcinoma, lipid-rich carcinoma, spindle cell carcinoma, and inflammatory carcinoma) [18,19]. Tubular carcinoma (adenocarcinoma) is the most common type of mammary gland tumor in dogs [19,20]. Mesenchymal neoplasms are sarcomas (osteosarcoma, fibrosarcoma, chondrosarcoma, liposarcoma, hemangiosarcoma, and others), with osteosarcoma as the most frequent mesenchymal neoplasm of the canine mammary glands [19]. However, some of them have mixed histology consisting of a combination of epithelial and myoepithelial or mesenchymal tissue (complex carcinoma, carcinosarcoma, and benign mixed tumors) [19]. Benign mammary tumors are mostly simple and complex adenomas, fibroadenomas, myoepithelioma, ductal adenoma and ductal papilloma [18,19].

The cytology of canine mammary tumors can be another approach in diagnostics, but it should be taken into account carefully, because it offers many false results due to lesions differentiation, atypic benign forms, and the presence of inflammation or necrosis in the tissue [21,22].

The Nottingham histological grade (NGS), described by Elston and Ellis in 1991 [23], is also used to provide prognostic information by assessing a malignancy score (nuclear pleomorphism, mitotic index, and tubule formation) [23]. In NHG, semi-quantitative evaluation of tubule formation (≥75% of tumor area containing tubules—score I; 10–75% of tumor area containing tubules—score II; ≤10% of tumor area containing tubules—score III), quantitative and qualitative judgement of nuclear polymorphism (small nuclei with regular outlines—score I; visible nucleoli and mild nuclei variability in size and shape—score II; severe nuclei variability in size and shape—score III), and mitotic count using a minimum 10 fields of tumor area (≤9 mitoses—score I; 10–19 mitoses—score II; ≥20 mitoses—score III) [23]. After summing these three component scores, grades from I to III are generated, wherein scores 3–5 indicate a low-grade (I) tumor, scores 6–7 an intermediate (II) tumor, and scores 8–9 a high-grade (III) tumor [23].

### 1.3. Comparative Oncology

CMC exhibit several similarities with HBC, mainly at the clinical, genetic, molecular, pathological, and etiological levels which are summarized in Table 1 [24]. The clinical correlation between CMC and HBC comprises the onset age, tumor incidence, hormonal etiology, and identical disease course [25]. Interestingly, the average onset age of mammary tumors in dogs (after 6 years) is comparable with the incidence of breast cancer in humans (after 40 years). In addition, the aspects affecting the clinical outcome, such as tumor size, clinical stage, metastasis, and lymph node invasion, are also comparable [26]. Approximately 50% of canine mammary carcinomas are reported to metastasize to regional lymph nodes and lungs, and eventually bones [27,28]. In their human counterpart, almost 20% of HBCs have a prognosis to develop metastatic lesions [29]. Among pathologic characteristics, canine and human tumors share features in a long-term oncogenic environment, intratumoral heterogeneity, and acquired treatment resistance (Table 1) [30]. Markers have the potential to predict the response to a certain anti-cancer treatment [e.g., estrogen and progesterone receptors, cytochrome P450 or antigen Ki-67 for breast cancer; *UGT1A1* gene encoding UDP-glucuronosyltransferase 1-1 enzyme or specific mutations of *K-RAS* (Kirsten rat sarcoma virus), for colorectal cancer; human epidermal growth factor receptor 2, HER2, for breast or gastric cancer; *c-KIT* gene encoding tyrosine-protein kinase KIT for gastric cancer; DNA excision repair protein ERCC1 or tumor protein p53 for lung cancer; reviewed in [31,32]] and provide cancer prognostic information [e.g., antigen Ki-67 or cyclin D1, cyclin E, matrix metalloproteinase-2 (MMP-2), protein p21, tumor protein p53, CD44 or E-cadherin for bladder cancer; beta tubulin for lung cancer; human epidermal growth factor receptor 3 (HER3) or inhibitor of growth protein 3 (ING3) for melanoma; carcinoembryonic antigen (CEA) for colorectal cancer; reviewed in [32]]. Although molecular markers are not routinely used in veterinary medicine, it is not surprising that biomarkers of HBC are also detectable in CMC (Table 1) [26,33]. Specifically, several molecular characteristics, such as up- or downregulation of adhesion molecules (E-cadherin; platelet endothelial cell adhesion molecule-1, PECAM-1; carcinoembryonic antigen, CEA; mucin 1), overexpression of growth factors (epidermal growth factor receptor, EGFR; vascular endothelial growth factor, VEGF; epidermal growth factor, EGF; insulin-like growth factor-1, IGF-1), low or high hormone expression (estrogen, progesterone, prolactin), increased expression of enzymes (metalloproteinase and cyclooxygenase), downregulation of tumor suppressor genes (cyclin-dependent kinase inhibitor 2A, *CDKN2A*; phosphatase and tensin homolog, *PTEN*; breast cancer gene 1, *BRCA1*; breast cancer gene 2, *BRCA2*; and tumor protein 53, *TP53*), upregulation of oncogenes (*K-RAS*; or mitogen-activated protein kinase, *MAPK*), and elevated production of various proteins (Ki-67 antigen; proliferating cell nuclear antigen, PCNA; von Willebrand factor VIII) in CMC mimic HBC (reviewed in [26,33]). A well-known fact is that mutations of *BRCA1* or *BRCA2* tumor suppressor genes contribute to the formation of mammary gland tumors [34]. While the hereditary mutations of these genes have been observed in humans, no analogous hereditary pattern has been described for mammary cancers in dogs, although Rivera et al. reported a familial risk for mammary cancer in English Springer Spaniel dogs [35]. Etiological factors for mammary cancer development, sex hormones and obesity, are as common for canines as for humans (Table 1). A body mass index (BMI) higher than 25 increased the risk of breast cancer by about 1.3–1.5-fold in women [36]. Similarly, obese dogs at one year of age were more likely to develop mammary tumors [13]. In this background, CMC has been suggested as a spontaneous translational research model to study HBC [37]. Moreover, dogs have several advantages as models of HBC over in vitro cell cultures (e.g., heterogeneity, presence of the immune system, lack of cross-contamination or cell line instability) and rodent models (e.g., genetic diversity, intact immune system, larger body size and blood volume, long-term monitoring, and the same environment as humans) [37,38]. Despite these similarities, CMC and HBC have some histological discrepancies [19,20,39]. For example, benign tumors are more prevalent in CMC than in HBC [20]. Further, mammary tumors with a mesenchymal origin (fibrosarcomas) and mixed histology (carcinosarcomas or complex carcinomas) are often diagnosed in female dogs and are very rare in humans [19]. Instead, the most common type of mammary tumors in humans is the invasive ductal carcinoma [39].

### 1.4. Treatment

Even though the improved health of dogs, as a result of better quality and more easily reachable veterinary care, treatment, and better nutrition, allows dogs to live longer, the incidence of cancer in dogs is constantly increasing [20]. Surgery consisting of the removal of the affected (cancerous) glands and local lymph nodes is currently the only efficient treatment and can be curative in many dogs [40,41]. In malignant cases, chemotherapy and radiotherapy are applied [33,42]. However, this aggressive approach is expensive and limited, with no definitive data [33,42]. No protocol for chemotherapy in female dogs affected by mammary gland tumors has thus far been standardized [41]. In addition, chemotherapy in dogs has not been proven to be as effective in the treatment of mammary gland cancers as it is in women [42,43]. Standard chemotherapeutics, such as docetaxel and doxorubicin, have not demonstrated dramatically improved overall survival times [44]. Thus, at this time, no effective systemic treatment options for dogs with mammary tumors are available. Because of the limited treatment possibilities, early diagnosis of cancer, evaluation of the cancer progression, and tumor response to chemotherapy can increase the survival of dog patients. Biomarkers represent a valuable tool in cancer research since they offer many applications, such as screening, differential diagnosis, prognosis determination, prediction to treatment, and disease progression monitoring [45,46].

## 2. MicroRNAs as Potent Biomarkers

A biomarker is generally defined as a quantifiable measure of a normal biological process or pathological process or as a response to a therapeutic administration [47]. In other words, a biomarker offers information about the actual condition of a living organism. Changes in biomarkers expression levels, concentrations or structure may indicate the onset, progression or regression of some disorder in the body [48]. Biomarkers can be represented by nucleic acids (DNA or RNA) [49], peptides [50], proteins [51], lipids [52] or metabolites [53].

MicroRNAs (miRNAs or miRs) are becoming potential non-invasive cellular and molecular biomarkers for the prediction, diagnosis, prognosis, and therapeutic targets for various types of cancers. Several studies have thus far confirmed the relevance of miRNAs in cancer-associated processes, including proliferation, differentiation, invasion, angiogenesis, metastasis, apoptosis, and drug resistance (reviewed in [54,55,56,57]).

### 2.1. Biogenesis and Function

**Figure 1:**
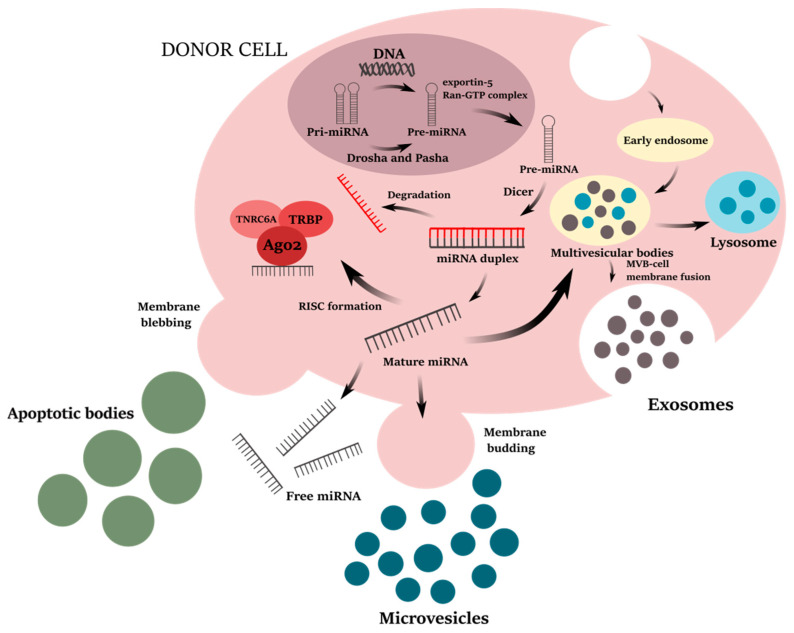
Biogenesis and release of microRNA (miRNA) and exosomes. The miRNA initially originates as primary miRNA (Pri-miRNA). Pri-miRNA is cleaved into the precursor miRNA (Pre-miRNA) by the Drosha enzyme and its cofactor Pasha [61,62]. Exportin-5 protein and Ran-GTP complex transport the pre-miRNA into the cytoplasm, where it is processed into the double-strand miRNA duplex by the action of a Dicer endonuclease [64,65,66]. One of the strands is degraded (so-called passenger strand; depicted with red color) and the second, mature miRNA strand (also known as guide strand; depicted with black color) is loaded into the RNA-induced silencing complex (RISC) by the binding to RNA-binding proteins (Argonaute 2, Ago2; trinucleotide repeat-containing gene 6A protein, TNRC6A; transactivation response RNA-binding protein, TRBP) [67,68]. The mature miRNA strand is then guided to the target messenger RNA (mRNA) to either degrade (perfect base complementarity) or inhibit the mRNA translation (partial base complementarity) [89]. The mature miRNA can be also secreted from the cell as free miRNA bound to RNA-binding proteins or incorporated, within the cell, into the extracellular vesicles (EVs), specifically exosomes and microvesicles [69,70]. Exosomes or small extracellular vesicles (sEVs; <200 nm) [90] are produced within the cells starting with the formation of early endosomes by cell membrane invagination [91,92,93]. The inner membrane budding of the early endosome leads to the maturation of the multivesicular bodies (MVBs) [91,92,93]. Some of MVBs are directed to lysosomes for degradation, while others are released to the extracellular space as exosomes after fusion with the plasma membrane [94,95]. Microvesicles or medium/large extracellular vesicles (m/lEVs; >200 nm–1000 nm) [90] are formed in the process of outward plasma membrane budding [96,97]. Apoptotic bodies (>1000 nm), the largest group of EVs, are released from the cells undergoing apoptosis by plasma membrane blebbing [90,98,99]. An original figure was created using Inkscape v1.1.2 software.

miRNAs are short (18–22 nucleotides), highly evolutionary conserved members of small non-coding RNAs discovered in 1993 in a model organism *Caenorhabditis elegans* [58,59]. The miRNA arises as a transcription product of non-coding regions or introns by RNA polymerase II [60]. Still in the nucleus, the resulting hundreds of nucleotides long primary miRNA (pri-miRNA) is subsequently cleaved by the endonuclease enzyme Drosha (RNAse III) and its cofactor microprocessor complex subunit DGCR8 (DiGeorge syndrome critical region gene 8, also known as Pasha), giving the precursor miRNA (pre-miRNA) [61]. Pre-miRNA has a hairpin and loop-shaped secondary structure with 80–100 nucleotides [62,63]. This pre-miRNA is transported from the nucleus into the cytoplasm by the exportin-5 protein and the Ran-GTP complex [64]. Here, the hairpin region of the pre-miRNA is processed by cytoplasmic ribonuclease Dicer into an 18 to 22 nucleotide long double-stranded miRNA duplex which contains two 5’ phosphorylated sequence strands with 3′ overhangs, named the mature miRNA guide strand and complementary passenger strand [65,66]. The miRNA duplex is unwinded into a single-stranded mature miRNA guide strand (depicted with black color in Figure 1), while the second passenger strand is degraded (depicted with red color in Figure 1) [67,68]. The mature miRNA strand binds to Argonaute 2 (Ago2) protein and other RNA-binding proteins (e.g., protein kinase RNA activator, PACT; trinucleotide repeat-containing gene 6A protein, TNRC6A; transactivation response RNA-binding protein, TRBP) to form an RNA-induced silencing complex (RISC) that regulates the translation of target messenger RNA (mRNA) [67]. In addition to the transcription repression within the cell, the mature miRNA can be also secreted from the cell as free (circulating) miRNA or intracellularly packed into the extracellular vesicles (EVs), such as exosomes (or small extracellular vesicles, sEVs) or microvesicles (or medium/large extracellular vesicles; m/lEVs) [69,70]. Mature miRNAs are selectively incorporated into the sEVs (exosomes) or enwrapped with microvesicles during their biogenesis (in process of early endosome inner membrane budding), as explained in Section 3.1, and subsequently, released to the extracellular milieu [70]. Such EV-packed miRNAs are delivered, through the EVs, to other target cells, where the miRNAs regulate their cognate target genes at the transcriptional level [69,71,72,73,74]. Within the cell, mature miRNAs are associated with RNA-binding proteins, such as Ago2, which protect free miRNAs from degradation by RNases after their release from the cells to the extracellular environment [75]. Free miRNAs are presented in different biofluids (such as blood plasma or serum [76,77], urine [78], breast milk [79], saliva [80], tears [81], or cerebrospinal fluid [82]) [75]. However, the precise mechanism of how free miRNA is released from cells is still not clear [69,70]. The process of miRNA biogenesis is summarized in Figure 1.

The miRNAs play a key role as negative post-transcriptional gene regulators in the safeguarding of all biological processes of multicellular organisms, including cell-cycle control, cell proliferation, differentiation, migration, metabolism, and apoptosis [83]. Regulatory action is mediated by the hybridization of miRNA to the 3′- or 5′-untranslated regions (UTRs) [84,85], or the open reading frame (ORF) [86] of the target mRNAs, resulting in the suppression of the expression of the protein-coding genes either by translational repression, mRNA degradation or both [87,88]. More specifically, perfect base complementary leads to mRNA degradation, while non-perfect (partial) base complementarity results in translation impairment [89].

### 2.2. The Role of miRNAs in Cancer

Since gene regulation at the transcriptomic level does not require the high complementarity of miRNA with the mRNA sequence, a single miRNA may target several mRNAs, and aberrant miRNA expression has the potential to considerably alter the expression level of several hundred transcripts [100,101]. Dysregulation of miRNAs is particularly prevalent in cancer, where the genetic instability of tumors (such as amplifications, deletions, mutations, epigenetic changes or polymorphisms) leads to altered miRNA expression profiles promoting oncogenesis [102,103]. Downregulated and deleted miR-15a and miR-16-1 in patients with chronic B-cell lymphocytic leukemia were firstly reported as altered miRNAs, leading to the onset, progression, and dissemination of cancer [104]. Subsequently, the interface between overexpression or ablation of miRNA and cancer development was exhibited in mouse models [102,105]. Nowadays, it is known that more than half of miRNAs are located in cancer-associated genomic regions [106]. Generally, miRNAs involved in cancer are either tumor suppressors or oncogenes, depending on the expression levels [107]. Overexpressed miRNAs, oncogenes, with a crucial role in the initiation and progression of cancer, have been termed oncomiRs [108]. As of February 2022, more than 40,000 free-full peer-reviewed articles dedicated to the investigation of the role of miRNA in cancer by diverse experimental approaches are available in the PubMed depository (https://pubmed.ncbi.nlm.nih.gov/?term=mirna+cancer&filter=simsearch2.ffrft (accessed on 1 February 2022)).

### 2.3. Non-Exosomal miRNA-Based Biomarkers of Canine Mammary Cancer

As of February 2022, 502 precursors and 453 mature miRNAs have been identified in the canine genome (miRBase database; https://www.mirbase.org/summary.shtml?org=cfa (accessed on 1 February 2022)) and most of them have been altered in CMC. As was discussed above, CMC and HBC demonstrate comparable clinical and pathological characteristics. Similarities in the miRNA expression pattern between canine mammary and human breast neoplasia have also been described [109] and several oncomiRs have been found to be highly conserved between dogs and humans [110,111]. These findings are not surprising, since dogs and humans share not only the same environment but also analogous diseases [112]. Moreover, considering the similarities between dogs and humans at the genetic level, miRNAs may target genes conserved between both. Aberrant expression of miRNAs implicated in cancer development, progression or metastasis may serve as a useful biomarker for diagnostic or prognostic purposes and, therefore, represent a target for therapy development [102].

Here, we review the most relevant miRNAs not packed into exosomes (hereinafter called non-exosomal) found in CMC studies in relation to the biomarkers for future clinical applications and compared their incidence in HBC. We found 11 articles related to the topic. According to the analysis carried out for this review, here the term “non-exosomal” is referred to miRNAs which come from sources such as mammary tumors [110,111,113,114,115], tumor mammary cell lines [116,117,118] or canine blood serum [119,120,121] using commercial kits.

The first study of miRNAs expression in CMC from 2008 investigated the expression levels of HBC key miRNAs (miR-15a, miR-16, miR-17-5p, miR-21, miR-29b, miR-125b, miR-145, miR-155, miR-181b, let-7f) in relation to CMC. Boggs et al. revealed that, apart from miR-145, the monitored miRNAs proved to have the same expression pattern as observed in humans [110]. The miR-15a and miR-16 show a significant downregulation in canine ductal carcinomas, while miR-21, miR-29b, miR-181b, and let-7f were upregulated in tubular papillary carcinomas. Mainly, miR-21 and miR-29b demonstrated statistically significant (*p* < 0.05) upregulation in canine tumor samples [110].

#### 2.3.1. miR-21

It is assumed that overexpression of miR-21 is a hallmark of carcinogenic cells and may serve as a common signal of pathological growth or cell stress [122]. The miR-21 is highly conserved and one of the most abundant miRNAs expressed in multiple mammalian cell types [122,123]. Physiologically, miR-21 regulates processes connected to cell growth, migration, and invasion [124]. In carcinogenesis, miR-21 acts as the oncomiR through the inhibition of tumor cell apoptosis [110,125,126]. Except in the study of Boggs et al. [110], the upregulated expression of miR-21 in canine benign or malignant tumors in comparison to normal glands was observed in several canine mammary studies [113,114,115,119,120]. The elevated expression of miR-21 in female dogs with mammary tumors is in correlation with progressive clinical stage and poor prognosis [119]. Thus, the level of miR-21 expression may be useful for distinguishing between bitches with mammary tumors (benign or malignant) and healthy ones (without mammary tumors) [119]. Moreover, increased expression of miR-21 in metastasis carcinoma (5.05-fold) compared to normal mammary gland makes it a good metastasis biomarker [114]. Regarding HBC, the altered expression of miR-21 was associated with increased cell proliferation, colony formation, migration, invasion, metastasis, angiogenesis, advanced tumor stage, lymph node metastasis, and poor patient survival [127,128,129,130,131,132]. Blocking miR-21 expression inhibits tumor growth and metastasis [133]. As miR-21 is one of the most upregulated miRNAs in HBC, it was postulated that targeting miR-21 by miR-21 inhibitors (anti-miR-21) as post-transcriptional gene silencing agents may have a therapeutic potential [134,135,136]. It follows that miR-21 represents a sensitive non-invasive biomarker for cancer screening, progression, and detection in CMC as well as in HBC.

#### 2.3.2. miR-29b

Another non-invasive biomarker for diagnostic and prognostic purposes for various types of cancer, including mammary cancer, can be miR-29b [137,138]. As a member of the miR-29 family together with miR-29a and miR-29c, miR-29b appears to have a crucial effect on mammary tumors by regulating multiple cancer-related processes essential for tumor development, such as proliferation, apoptosis, metastasis, fibrosis, angiogenesis, proteolysis or collagen remodeling [139]. However, the exact role of miR-29b in cancer remains controversial, as it has been declared as an oncomiR and tumor-suppressor [138,139,140,141,142]. The differential expression of miR-29b has also been noted in CMC. Together with the study of Boggs et al. [110], the upregulated expression of miR-29b was observed in canine SNP cell line (4.0714-fold) [116] or serum samples from canine mammary carcinoma dogs (2.78-fold) [121]. In contrast, a significant downregulation of miR-29b expression in metastasizing and non-metastasizing mammary tumors was observed in the studies of Jain et al. [119], Bulkowska et al. [113], and von Deetzen et al. [114]. Due to the altered expression of miR-29b in a metastatic group in comparison with benign tumors, miR-29b may present another valuable biomarker for metastasis [113]. An inconsistent downregulated [143] or upregulated [144] expression pattern of miR-29b was also observed in HBC, wherein this was connected with proliferation, migration, impaired apoptosis, increased tumor cell migration, and invasion.

#### 2.3.3. miR-141

The very first evidence of comprehensive expression profiles of the 277 investigated miRNAs from the canine genome, which were evaluated using a quantitative polymerase chain reaction strategy in cell lines derived from female dogs of different breeds with spontaneous mammary carcinomas or adenocarcinomas (CMT12, CMT27, and CMT28), revealed miR-141 to be a potent oncomiR [117]. In this study, miRNA-141, a member of the miR-200 family, was experimentally validated to target 3′-UTR of a tumor suppressor INK4 (inhibitor of CDK4), a member of the INK4/CDNK2 family of tumor suppressor genes, through the direct correlation between the overexpression of miR-141 and the target mRNA p16/INK4A in cell lines CMT12 and CMT27 [117]. Significant high expression levels of miR-141 are strongly associated with highly aggressive breast carcinomas (grade III) when compared to grade II breast cancer. ROC curve analysis revealed the diagnostic and prognostic utility of miR-141 in the discrimination of malignant from benign breast tissues (ROC-AUC = 0.97). Moreover, high expression of miR-141 is associated with worse overall survival (OS) in breast cancer patients (HR = 1.43, 95% CI = 1.17–1.74, *p* = 0.00037; among 1262 patients) [145]. Additionally, upregulation of miR-141 promotes the migratory and invasive abilities of an aggressive triple-negative breast cancer cell line MDA-MB-231 through regulation of the phosphatidylinositol-4,5-bisphosphate 3-kinase/protein kinase B (PI3K/AKT) signaling pathway by increased secretion of vascular endothelial growth factor A (VEGF-A) and expression of integrin-αV [146]. Together, all these data highlight the role of miR-141 as a valuable biomarker with potential clinical applications in CMC as well as HBC.

#### 2.3.4. miR-429 and miR-200c

The study of Lutful Kabir et al. reported another group of miRNAs to be altered in both canine mammary and human breast tumors [117]. The miR-9, miR-155, miR-200a/b, and miR-429 were overexpressed, whereas miR-1, miR-133a/b/c or miR-214 were found to be downregulated in canine cell lines CMT12, CMT27, and CMT28 [117]. In particular, miR-429 and miR-200c were found to be highly upregulated (>1000 fold and 100–150 fold, respectively) and predicted to target the tumor suppressor ERBB receptor feedback inhibitor 1 (ERRFI1) mRNA [117]. Thus, both miRNAs act as oncomiRs in CMC [117]. Comparable to HBC, miR-429 was also described as an oncomiR that affects the hypoxia-inducible factor 1-alpha (HIF1α) pathway by targeting VHL mRNA [147]. The overexpressed miR-429 in breast cancers with amplified human epidermal growth factor receptor 2 (HER2+) was responsible for the increased proliferation and migration of breast cancer cells, while the silencing of miR-429 had an impact on tumor growth postponement [147]. In contrast, miR-200c was reported as a tumor suppressor in breast cancer tissue and cell lines where suppress the cell proliferation by targeting KRAS mRNA [148], contributes to the paclitaxel resistance by targeting (sex-determining region Y)-box 2 (SOX2) transcriptional factor [149], or inhibits the metastasis of triple-negative breast cancer [150]. Since both miRNAs are involved in the tumorigenesis and progression of a variety of cancers, they may represent potent biomarkers in CMC and HBC.

#### 2.3.5. miR-497

Tumor-suppressor miR-497 family members (miR-497, miR-195, miR-15, and miR-16) were found to be downregulated in canine mammary cell lines [118]. Downregulation of miR-497 was also observed in the CMT1211 and CMT7364 cell lines compared to primary canine mammary gland cells [118]. Transfection of miR-497 mimic and inhibitor into the canine mammary tumor cells showed that overexpression of miR-497 significantly inhibited cell proliferation and migration, and increased the apoptosis in the CMT1211 and CMT7364 cell lines [118]. The observed negative correlation between miR-497 and the expression of interleukin-1 receptor-associated kinase-like 2 (IRAK2) suggested *IRAK2* as a functional target gene of miR-497. The suppression of IRAK2 mRNA by the overexpressed miR-497 induced apoptosis by inhibiting the activation of the pro-survival NF-ĸB (nuclear factor kappa-light-chain-enhancer of activated B cells) pathway [118]. This study demonstrated that miR-497 inhibits cancer cell growth, with the suggestion of the miR-497/IRAK2/NF-ĸB axis as a potential mechanism for CMC development [118]. Therefore, miR-497 was suggested as a diagnostic biomarker and therapeutic target in CMC [118]. These findings are consistent with HBC, where miR-497 was among the most prominently downregulated miRNAs [151]. Several studies have demonstrated that overexpression of miR-497 inhibited the proliferation, invasion, metastasis, angiogenesis or cell cycle of cancer cells, and induced apoptosis in HBC by targeting Bcl-2-like protein 2 (Bcl-w) [152], B-cell lymphoma 2 protein (Bcl-2) [153], yes-associated protein 1 (YAP1) [154], HIF-1α [155], or cyclin E1 [156] mRNA.

#### 2.3.6. miR-10b, miR-101, miR-125a/b, miR-136, miR-143, miR-145, let-7f, and miR-203

Several miRNAs demonstrated a more important role in the metastasis process than in the malignant transformation. Downregulated miR-10b, miR-101, miR-125a/b, miR-136, miR-143, miR-145, and let-7f, as well as upregulated miR-203 were found in a metastatic group in comparison with non-metastasizing or benign canine mammary tumors [113,114]. The expression levels of miR-10b, miR-125b, miR-136, and let-7f in particular gradually decreased from normal mammary tissue, through benign tumors and non-metastatic malignant tumors, to metastatic tumors [113]. These findings are of great predictive importance for the course of a disease and, therefore, altered miRNAs may constitute molecular markers of metastasis.

On the other hand, the expression level of miR-143 in non-metastasizing mammary carcinoma [114] or the canine SNP cell line established by Osaki et al. [116] was higher in comparison to normal mammary gland tissue (2.70-fold and 1547.9-fold, respectively). Likewise, miR-203 expression was downregulated in benign tumors compared to a healthy control group [113]. Such discrepancies in the expression level of one particular miRNA may be a result of changes in gene expression in the tumor, different tumor phenotypes or even different data analyses used to evaluate miRNA expression [113].

#### 2.3.7. miR-210

Some miRNAs are expressed at different stages of malignancy [114]. For example, miR-210 was found to be present in malignancies, such as adenoma, non-metastasizing carcinoma, metastasizing carcinoma, and metastatic tissue with gradually increased expression (7.01-fold, 10.41-fold, 10.72-fold, and 19.63-fold respectively) [114]. As explained by the authors of the study, miR-210 has been termed a hypoxamir due to its upregulation as a result of hypoxia in tissues and it mediates the metabolic adaptation to anaerobic conditions [114,157]. Therefore, rising expression during the progression of malignancy may be a result of increased hypoxia in tumor growth. Since miR-210 is associated with the formation of capillary-like structures [158], the author also hypothesized its role in metastasis by enhanced angiogenesis. This makes miR-210 another potential diagnostic marker in malignancies [114]. Higher expression of miR-210 in canine neoplasms than in a control group was also observed in the study of Bulkowska et al. [113]. In HBC tissue, overexpression of miR-210 correlates with lymph node metastasis, clinical staging, differentiation and poor prognosis in patients with breast cancer. Therefore, miR-210 was proposed as a potential prognostic biomarker of breast cancer [159,160].

#### 2.3.8. miR-138a

Among 18 significantly decreased miRNAs in the canine SNP cell line, miR-138a showed the greatest reduction in the expression (0.007-fold) [116]. As discussed in this study, tumor-suppressive miRNA-138a represses the epithelial-mesenchymal transition (EMT), a process resulting in cancer aggressiveness and metastasis. Since this study showed that some SNP cells were positive for vimentin as an important EMT marker [161], the authors declared that SNP cells undergo the EMT process, which also confirms the suppressive and biomarker role of miR-138a in CMC [116].

#### 2.3.9. miR-8832, miR-96, and miR-149

Genome-wide methylation profiling in canine mammary tumors revealed miR-8832 as a new miRNA associated with both CMC and HBC [111]. Downregulated *GNAO1* (guanine nucleotide-binding protein-alpha O1) in canine mammary tumors was predicted as its target gene. As discussed by the authors, this tumor suppressor gene is involved in the reduction of cell proliferation in some human cancers, and dysregulation of *GNAO1* mRNA may be involved in tumorigenesis. Thus, miR-8832 represents a potential biomarker in both canines and humans [111].

The study also identified other miRNA candidates, upregulated miR-96 and downregulated miR-149, reported as cancer-associated miRNAs in humans [111]. Oncogenic miR-96 was found to be constantly upregulated in breast cancer tissues where it promotes proliferation, migration, and the invasion of cancer cells through silencing the target gene *PTPN9* (gene for tyrosine-protein phosphatase non-receptor type 9) [162]. Tumor-suppressive miR-149 contributes to breast tumor progression by supporting aberrant Rac activation [163] and recruitment of macrophages to the tumor [164]. Using the sequence-based target prediction program TargetScan, the authors predicted *BRPF3* (gene encoding a bromodomain and PHD finger containing 3), *ADCY6* (gene encoding adenylyl cyclase type 6), and *LRIG1* (gene encoding leucine-rich repeats and immunoglobulin-like domains protein 1) as targets for miR-96, and RNF2 (gene encoding E3 ubiquitin-protein ligase RING2) as a target for miR-149, highly conserved genes in dogs and humans [111].

Generally, miRNAs are more stable (up to 10-times) than mRNAs [165,166] and easy to detect in samples, such as tissues obtained from biopsy or surgery or biological fluids (such as serum, plasma, urine, saliva, seminal, ascites, amniotic pleural effusions, or cerebrospinal fluid) [167]. However, invasive procedures, such as tissue sample collection, are not very suitable for diagnostic or screening purposes, as mammary biopsies may yield a very small amount of RNA, with differences in quantified miRNAs at the level of one nucleotide [110]. In this regard, feasible and relatively non-invasive biofluid-extracted circulating miRNAs have attracted interest in the term of biomarkers as novel diagnostic tools for cancer, as this would limit the need for the collection of tissue samples and other invasive procedures [168]. Except for simple isolation, circulating miRNAs maintain stability under different conditions of sample processing and isolation [169]. Circulating miRNAs, as well as intracellular miRNAs, are also involved in the regulation of several biological processes with abnormal expression during pathological conditions [170]. Altered expression of circulating miRNAs is related to the initiation and progression of cancer [170]. Biofluid miRNAs show dynamic changes in physiological and pathological states before the clinical signs appear [171]. Furthermore, importantly, circulating miRNAs can be easily detected by basic molecular techniques [170]. Several circulating miRNAs have been described as biomarkers in cancer, including HBC (reviewed in [170,172]). Based on a literature review, we found four studies investigating levels of circulating miRNAs in plasma or serum samples in canine mammary tumors [113,119,120,121]. Nonetheless, the first study by Bulkowska et al., comparing differences between metastatic and non-metastatic tumors, showed no significant differences in the expression of selected metastasis-specific miRNAs (cfa-miR-144, cfa-miR-32, cfa-miR-374a, and hsa-miR-1246) by polymerase chain reaction (PCR) analysis [113]. On the other hand, the recent study by Fish et al. revealed circulating miRNAs as biomarkers of canine mammary carcinoma [121]. In this work, serum miRNA from 10 healthy female dogs and 10 bitches with histologically confirmed mammary carcinoma revealed 452 unique serum miRNAs by RNA deep-sequencing and 65 miRNAs differentially expressed (>±1.5-fold) and statistically significant between groups (carcinoma vs. healthy) by digital droplet PCR (dPCR). Although the expression of several miRNAs, such as miR-29b, miR-34c, miR-122, miR-125a, and miR-181a, was found to be upregulated, the authors suggested differentially expressed circulating miR-18a and miR-19b as the most potential biomarkers.

#### 2.3.10. Circulating miR-18a

Significantly upregulated serum miR-18a (1.94-fold by RNA sequencing; 1.24-fold by dPCR) was suggested as a candidate prognosis biomarker for CMC [121]. The authors revealed significantly higher levels of miR-18a in the group with histologic evidence of lymphatic metastasis invasion than without (2.82 versus 1.23 reads per million). Thus, miR-18a was proposed as a strong candidate prognostic biomarker also for HBC risk [121]. Circulating miR-18a was also overexpressed in a set of 60 serum samples from women with early-stage breast cancer compared to a sample of 51 healthy controls, suggesting miR-18a as a blood-based multi-marker for the early detection of HBC [173]. Generally, miR-18a, a member of the miR-17-92 cluster, suppresses the translation of estrogen receptor α (ERα), thus decreasing the protective effect of estrogen [174]. This finding was also observed in breast cancer-derived cell lines MCF-7 and MDA-MB-231, wherein not only the low expression of the ER, but also a decreased sensitivity to tamoxifen, and endocrine resistance, was associated with miR-18a high expression [175]. In another study, the overexpression of miR-18a in breast cancer cell lines MCF7 and ZR-75-1 led to an increase in the cells’ proliferation and migration, significant repression of E-cadherin, activation of genes of the Wnt (Wingless and Int-1) noncanonical pathway, PCP (planar cell polarity) pathway, JNK (c-Jun N-terminal Kinase) pathway, and actin remodeling [176]. Furthermore, miR-18a was suggested as an early driver of tumorigenesis, since it was found to be upregulated in contralateral unaffected breasts and benign biopsy samples before the development of breast cancer [177].

#### 2.3.11. Circulating miR-19b

Another significantly upregulated (3.15-fold by RNA sequencing; 1.76-fold by dPCR) serum miR-19b was proposed as a candidate diagnostic biomarker [121]. The ability to distinguish between mammary tumor-bearing dogs and dogs without neoplasia based on miR-19b was also revealed in this study with the ROC-AUC (receiver operator characteristic-area under the curve) and sensitivity/specificity analysis (ROC-AUC = 0.978) [121]. The miR-19b is a key molecule for cancer development, as it was found to be an active participant in the pathogenesis of various types of cancer, including HBC [178,179]. In breast cancer studies, miR-19b has demonstrated tumor-promoting activities. The wound-healing assay and transwell invasion assay performed by Zhao et al. demonstrated that overexpressed miR-19b facilitated the migration and metastasis of breast cancer cells by downregulation of myosin regulatory light chain interacting protein (MYLIP) involved in the regulation of cell movement and migration [179]. In the same study, miR-19b promoted the downregulation of E-cadherin and upregulation of intercellular adhesion molecule 1 (ICAM-1), and Integrin β1 in vitro and in vivo, leading to the activation of downstream signaling pathways (the Ras-MAPK pathway and the PI3K/AKT pathway) and involved genes [179]. In another study, miR-19b was found in less invasive breast lines (MCF-7, T47D, and ZR-75-1 cells) as well as in invasive breast lines (MDA-MB-231 and BT-20 cells), wherein it regulated at a post-transcriptional level the expression of tissue factor, known as a regulator of tumor angiogenesis and metastasis [178]. Taking together the results of these studies, miR-19b serves as an oncomiR in the progression of breast cancer and could act as a biomarker.

#### 2.3.12. Circulating miR-21 and miR-29b

The latest studies from 2021 investigated serum miRNA-based biomarkers, miR-21 and miR-29b. Both miRNAs were also altered in tumor samples, as discussed above. In the study of Jain et al., serum samples of 60 female dogs (20 healthy/control, 20 with benign tumors, and 20 with malignant mammary tumors) were used [119]. Serum miR-21 was upregulated in malignant (3.0-fold) and benign (1.8-fold) tumors compared to the control samples (1.1-fold), while the expression of serum miR-29b was significantly downregulated in the malignant and benign group compared to the control samples (0.2-fold, 0.4-fold, and 1.1-fold, respectively). Interestingly, the expression was higher/lower in malignant tumors than in benign tumors. As suggested by the authors, circulating miR-21 could serve as a prognostic marker for the early detection of canine mammary tumors, and miR-29b can add sensitivity and accuracy to a diagnosis if evaluated together with miR-21 [119]. In the study of Ramadan et al., miR-21 was significantly upregulated (12.84-fold) in the serum samples of 10 female dogs with mammary tumors compared to the control group of 7 healthy bitches. Thus, miR-21 was hypothesized as a more sensitive, non-invasive indicator for CMC [120]. These observations are in accordance with other studies on tumor samples [110,113,114].

Despite the above-mentioned advantages of circulating miRNAs as biomarkers (non- or minimally invasive availability and easy accessibility, stability or resistance toward severe stressing conditions, such as high temperatures, repeated freeze–thaw cycles), they still have several issues hindering their reliability for the clinical application [180]. One of the major limitations of circulating miRNAs as biomarkers is the inability to identify their exact origin [181]. For example, most circulating miRNAs are obtained from blood using plasma or serum as the source [181,182]. However, blood contains a variety of cell types that challenge the identification of the cell origin of a particular miRNA [181]. The majority of the miRNAs in the blood are packaged in EVs like microvesicles (or m/lEVs) and exosomes (or sEVs) [180]. Exosomes and exosome-derived miRNAs have attracted great attention in recent years in terms of biomarkers [183]. A literature review of miRNAs from exosomal and non-exosomal sources showed that 71% of the selected articles concluded that exosomes are the source of choice for miRNAs in biomarker studies. In addition, 75% of articles comparing both sources of miRNAs recommended exosome-derived miRNAs over non-exosomal miRNAs [181]. Thus, it can be assumed that exosomes can be a better source of miRNAs as biomarkers due to their benefits in terms of quantity, quality, and stability [181], as discussed below.

## 3. Exosomes

### 3.1. Nomenclature

The International Society for Extracellular Vesicles (ISEV) approves the definition of EVs as lipid bilayer-surrounded particles released from the cell without the ability to replicate. Due to intersecting characteristics and the lack of consensus on specific markers of different EV subtypes (e.g., expression of CD9, probable marker of exosomes and ectosomes; [184]), some authors suggested rather to consider the origin of EVs. Based on this, the term exosomes should refer to the intracellular compartment-originated EVs and ectosomes (microparticles/microvesicles) as EVs derived from the plasma membrane [185,186]. However, the EVs’ designation to a particular biogenesis pathway is challenging. Therefore, the ISEV proposed in 2018 “Minimal information for studies of extracellular vesicles 2018 (MISEV2018): a position statement of the International Society for Extracellular Vesicles and update of the MISEV2014 guidelines” as recommendations for EVs nomenclature [90]. In total, 94% of MISEV2018 respondents affirm the classification of EVs subtypes according to either (i) physical characteristics such as size (“small EVs”; sEVs (<100 nm or <200 nm) and “medium/large EVs”; m/lEVs (>200 nm)) or density (low, middle, high), (ii) biochemical composition of surface markers (e.g., CD63+ EVs, CD81+ EVs, CD81− EVs, CD9+ EVs) or (iii) origin of parental cell or biological processes (e.g., tolerosomes, oncosomes, apoptotic bodies) [90]. However, the reviewed literature does not take into account the MISEV2018 guidelines and keeps the term “exosomes”. To avoid misunderstanding in this review we decided to keep both terms “exosomes” and “sEVs”.

### 3.2. Biogenesis

**Figure 2:**
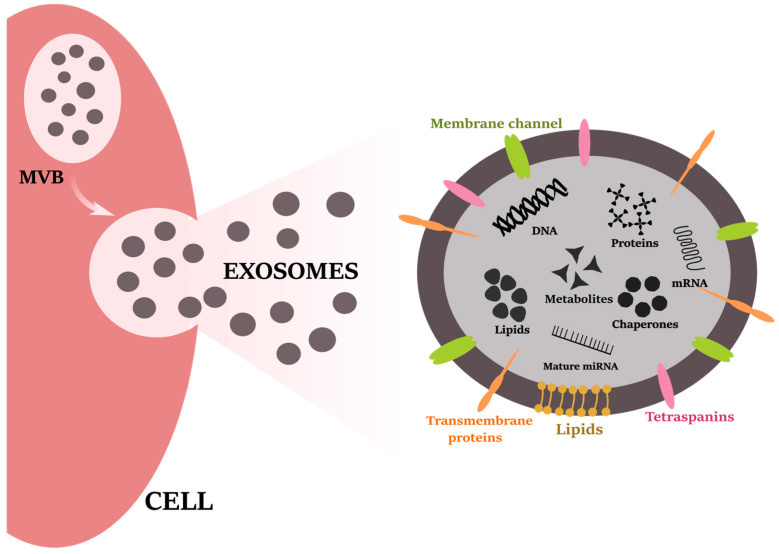
Exosomal cargo. During the process of budding of the inner membrane of the early endosome, some proteins are incorporated into the invaginating membrane, and the cytosolic components are enclosed inside [198]. sEVs (exosomes) contain selective repertoires of proteins, DNA, messenger RNA (mRNA), non-coding RNAs (miRNA), lipids, and metabolites that moderate signaling pathways in the recipient cells [71,199]. Tetraspanins (CD9, CD63, CD81, and CD82) or chaperones (HSP70, HSC70, and HSP90) represent exosomal markers [211,212]. Biologically active cargo of sEVs (exosomes) participates in several physiological or pathological processes, including cancer. An original figure. The figure was created using Inkscape v1.1.2 software.

Since the identification of exosomes in sheep reticulocytes in the 1980s [187,188], these small endosomal-derived membrane vesicles have gained high interest over the last decade. sEVs (exosomes) are a subset of EVs secreted into the extracellular space by prokaryotic and eukaryotic cells, as well as in physiological and pathological processes [189]. To distinguish them from other EVs excluded from the body fluids, Rose Johnstone and colleagues gave them the name exosomes [190], now called sEVs based on the MISEV2018 guidelines [90]. As was described above, EVs are generally categorized based on their size into sEVs or m/lEVs [90]. Microvesicles (also known as ectosomes, microparticles or m/lEVs) have typically a diameter of medium/large-sized EVs (>200 nm–1000 nm) and are formed in the process of outward plasma membrane budding [90,96,97]. The suggested protein markers are CD40, selectins, and integrins [191]. Whereas sEVs (exosomes) and m/lEVs (microvesicles) are secreted during normal cellular processes, apoptotic bodies (>1000 nm) are only formed and released from the cells undergoing programmed death by plasma membrane blebbing [98,99] and express phosphatidylserine, the so-called “find-me, eat me” signal that triggers macrophage clearance [192,193,194]. Apoptotic bodies differ from the other two major EV groups by containing fragments of host DNA and cellular organelles [193]. These EVs can be distinguished by protein markers, such as histones, thrombospondins, and C3b [195]. sEVs (exosomes) are nano-sized (<200 nm) EVs surrounded by a lipid bilayer membrane which is characteristic for all EVs and protects the encapsulated material, such as nucleic acids (DNA, mRNA, and non-coding RNAs), proteins, peptides, chaperons, lipids, metabolites, from the extracellular environment (Figure 2) [191,196]. Other authors subclassify the sEVs (exosomes) based on the size into exomeres (35 nm), small exosomes (Exo-S) (60–80 nm), and large exosomes (Exo-L) (>90 nm) [197]. sEVs (exosomes) are produced within the cells by an endocytic pathway regulated by proteins and lipids in form of the multivesicular bodies (MVBs) and released to the intercellular space after fusion with the cell membrane [94]. Shortly, sEV (exosome) maturation begins with the formation of the early secretory endosome mediated by clathrin- or caveolin-dependent or independent invagination of the cell membrane, together with the accumulation of bioactive substances [91,92,93]. The budding of the inner membrane of early endosomes leads to the maturation of the MVBs [91,92,93]. During this process, some proteins are incorporated into the invaginating membrane, while the cytosolic components (such as nucleic acids, protein, chaperones, peptides, metabolites, and lipids) are enclosed inside (Figure 2) [198]. MVBs are late endosomes containing intraluminal vesicles. MVBs are of two destinies: (1) direction to the lysosome for degradation by enzymes in the lysosome lumen; or (2) fusion with the plasma membrane to release the content (i.e., intraluminal vesicles) into the intercellular space (Figure 1) [94,95]. The factors determining the direction of MVBs are still poorly known [199]. However, it was found that secreted MVBs contain an important pool of cholesterol [200]. This observation raises the question of whether high levels of cholesterol may be the determining parameter of MVBs’ destiny. Most of the released intraluminal vesicles are sEVs (exosomes). The biogenesis of MVBs, together with exosome formation and release, is mediated by endosomal sorting complexes required for transport (ESCRT) mechanism and other ESCRT-associated proteins (vesicle trafficking 1, VTA1; apoptosis-linked gene 2-interacting protein X, ALIX; tumor susceptibility gene 101 protein, TSG101; or vacuolar protein sorting-associated protein; VPS4) [201,202,203]. The ESCRT is complex machinery that comprises four different types of multiprotein sub-unit complexes, named ESCRT-0 to III. ESCRT-0 is responsible for the recognition and recruitment of ubiquitinated cargo to the endosomal membrane, ESCRT-I and II for the membrane budding, and ESCRT-III mediates vesicle separation from the plasma membrane [204]. Additionally, recent evidence has demonstrated the effect of ESCRT-independent pathways on exosome formation [205,206,207]. It can be assumed that exosome formation is controlled by factors in the cell and tissue microenvironment [97,208,209,210]. On the one hand, the production of sEVs (exosomes) is cell-regulated, as needed [97]. On the other hand, cell stress factors (hypoxia, acidosis) [208,209] or stimulation by growth factors (epidermal growth factor) [210] were found to induce exosome production and exocytosis. Several protein markers, including tetraspanins (CD9, CD63, CD81, and CD82), ALIX, TSG101, flotillin, heat shock proteins (HSP70, HSC70, HSP90), and T-complex protein 1 subunit beta (CCT2), are suggested as markers to differentiate sEVs (exosomes) from other EVs [211,212], even though they are recognizable by electron microscopy thanks to their typical biconcave or cup-like shape.

### 3.3. Function, Isolation, and Storage

Initially, sEVs (exosomes) were considered to be cellular waste released after cell damage or as unnecessary products of cell homeostasis, with no significant function and impact on neighboring cells [213]. The important role of sEVs (exosomes) as actual mediators of physiological pathways was revealed 10 years after their discovery by Raposo et al. [199] and, later, a plethora of other studies. sEVs (exosomes) are ubiquitous in healthy or pathological conditions and found to be secreted in biofluids, such as urine [214], blood plasma and serum [215], breast milk [216], colostrum [217], amniotic fluid [218], tears [219], vitreous humor [220], aqueous humor [221], synovial fluid [222], saliva [223], and tumor ascites [224]. sEVs (exosomes) represent a novel route of cell-to-cell communication [225]. When reaching the target cells, sEVs (exosomes) release their complex cargo, represented by proteins, metabolites, lipids, DNA, RNA and small non-coding RNAs (including miRNAs) (Figure 2), which may eventually reprogram the recipient cells [71,199]. Thus, sEVs (exosomes) and their biologically active cargo may be important in a variety of physiological or pathological processes, including immune response [99,226,227], inflammation [228,229,230], signal transduction [231,232,233], angiogenesis [234,235,236], antigen presentation [237,238,239], neurodegenerative diseases [240,241,242], cardiovascular diseases [243,244,245], renal diseases [246,247,248], viral infection [249,250,251], pregnancy [252,253,254], cancer progression [255,256,257], and cell death [98,258,259].

To allow the application of sEVs (exosomes) as biomarkers, effective isolation methods and optimal storage conditions are crucial. The most commonly used method is ultracentrifugation, followed by ultrafiltration, differential centrifugation, microfluid-based techniques, immunoaffinity chromatography, the polyethylene glycol-based precipitation method, and size-exclusion chromatography [260]. Each technique has its pros and cons and differs in the processing of the sample and the purity and quality of the exosomes obtained (reviewed in [261,262]). Commercial kits are also available on the market, like exoEasy Maxi kit (Qiagen, Hilden, Germany), Total Exosome Isolation Kit (Invitrogen™), ExoQuick^®^ (System Biosciences, Palo Alto, CA, USA), MagCapture™ Exosome Isolation Kit PS (Wako, Richmond, VA, USA), Exosome Isolation Kit Pan (Miltenyi Biotec Inc., Cologne, Germany), Intact Exosome Purification (Norgen Biotek Corp., Thorold, ON, Canada) or Minute™ Hi-Efficiency Exosome Precipitation Reagent (Invent, Plymouth, MN, USA). Commercial kits are time-saving and less laborious. At the same time, kits are expensive, and several studies have demonstrated that different kits may introduce variations in the concentration, purity, and size of sEVs (exosomes) [263,264,265]. Thus, when evaluating results, it is necessary to take into account the advantages and disadvantages of individual isolation methods of EVs.

The great advantage of sEVs (exosomes) is the possibility of their long-term storage at lower temperatures before analysis, with no or minor impact on exosome yield or bioactivity [171,266]. However, storage temperature depends on the sEVs (exosomes) source. For example, urine exosomes are sensitive to the storage temperature [267]. Zhou et al. showed that storage of urine samples at −20 °C led to a significant loss of exosomes compared to freshly collected urine. Preservation at −80 °C combined with extensive vortexing after thawing maximized the efficiency of exosome recovery [267]. On the other hand, multiple studies have shown that blood components, such as plasma or serum, can be stored long-term (for several years) either at 4 °C, −20 °C or −80 °C, and even at room temperature for short time (1–2 days), with no significant exosome or exosome-associated RNA and proteins degradation [268,269,270,271,272]. However, the study of Dutta et al. showed a decrease in central nervous system-derived α-synuclein stability upon storing serum or plasma-originated exosomes after 5 years at −80 °C [273].

To summarize, the ability of exosomes to transfer regulatory messages to other cells and their availability and stability make them a valuable source of biomarkers.

### 3.4. Exosome-Derived miRNAs as Biomarkers

Nowadays, sEVs (exosomes) are of interest in biomarker research. Naturally, this raises the question of why exactly sEVs (exosomes)? Exosome cargo (represented by nucleic acids, proteins, peptides, lipids, and metabolites; Figure 2) is specific and may vastly differ among various cell types, even from the same primary cell [274], depending on their function and current state (e.g., normal, transformed, differentiated, stimulated, and stressed). Thus, cell- or condition-specific sEV (exosome) content is something like a fingerprint of the donor cell reflecting the cellular processes and, therefore, may serve as biomarkers for various diseases [213]. Principally, the demonstration of miRNAs association with EVs by Valadi et al. in 2007 [71] open the way for a multitude of studies dealing with EV-associated miRNAs. Exosome-derived miRNAs have attracted considerable attention as non-invasive biomarkers of various diseases with diagnostic and prognostic potential [183,275,276]. To describe selectively packaged, secreted, and transferred miRNAs between cells in sEVs (exosomes) and distinguish them from circulating miRNAs, Bhome et al. introduced the term “exomiRs” [277]. These exomiRs offer some beneficial factors over circulating miRNAs that increased their importance as biomarkers. Except for the above-mentioned fact that the miRNA profile presents a signature of the parental cell, sEVs (exosomes)-packaged miRNAs are highly protected from degradation, even in non-optimal storage conditions and in the presence of RNases, hence conditions that normally degrade free miRNAs [277,278,279]. Indeed, sEVs (exosomes) are considered to be a stable source of miRNAs, and exosomal miRNAs in biofluids are more stable in comparison to circulating miRNAs [280]. ExomiRs have been shown to maintain stability either for short-term storage (2 weeks) at 4 °C or long-term storage (5 years) at −20 °C, as well as resistance to freeze–thaw cycles [171]. Due to their ease of access and stability, exomiRs represent a minimally invasive tool for the diagnosis and prognosis of cancer. The fact that exomiRs are also secreted by other cell types and not only cancer cells could mask cancer-specific biomarkers [278]. Profiling multiple exomiRs markers and isolating exosomes using tumor-specific protein markers could improve exosomal miRNAs sensitivity and specificity [278].

Today, research generally monitors and measures miRNAs, as well as exomiRs, using microarrays and real-time PCR (RT-PCR) [275]. Microarrays can detect many aberrant miRNAs with the entire genome expression profiling of miRNAs in the sample, but without determination of absolute quantification [275,281]. Being more sensitive and specific, RT-PCR allows the detection of low-level miRNAs with the determination of absolute quantification [275,281]. However, it cannot be used to identify novel miRNAs [281]. Novel miRNAs and miRNAs distinguished only by one nucleotide can be detected by the accurate and sensitive method of RNA sequencing because no primers or probes are needed [275,281]. RNA sequencing was already applied in the detection of exosomal miRNAs [282,283,284].

### 3.5. ExomiRs in Canine Mammary Cancer

Cancer cell-derived sEVs (exosomes) are not only inert cellular by-products but are, indeed, functionally and biologically important in neoplastic transformation [285] and/or tumor progression (reviewed in [205,286]). Cancer cells have been found to secrete more sEVs (exosomes) than non-malignant cells [287,288,289,290,291]. For instance, the concentration of exosomes quantified by Exotest (author-designed ELISA) using CD63 and caveolin-1 as detection antigens was significantly (*p* < 0.001) higher in melanoma patients with respect to healthy [289]. More production of sEVs (exosomes) (quantified using nanoparticle tracking analysis and expression of the suggested markers of sEVs (exosomes)—Alix and TSG101) by B42 clone 16 breast cancer cell line [(53.2 ± 1.6) × 10^8^ exosomes per 10^6^ cells] compared to normal mammary epithelial cells HMEC B42 [(4.5 ± 2.3) × 10^8^ exosomes per 10^6^ cells] was demonstrated by Riches et al. [288]. A two-fold increase in the number of sEVs (exosomes) (with sizes in the range 85–150 nm confirmed using nanoparticle tracking analysis) from plasma (13.3 × 10^11^ particles/mL) and serum (9.9 × 10^11^ particles/mL) of human prostate cancer patients in comparison to the healthy control (4.15 × 10^11^ particles/mL) indicate that tumor cells produce more sEVs (exosomes) than the normal cells [287]. The relative concentration of circulating sEVs (exosomes) with confirmed a lipid bilayer and CD9 positivity was significantly higher (*p* < 0.05) also in the sera of patients with pancreatic ductal adenocarcinoma compared to healthy donors [290]. Additionally, significantly (*p* < 0.001) greater levels of sEVs (exosomes) with vesicular structures and size ≤ 100 nm confirmed by electron microscopy associated with three stages (I, II, and III) of ovarian cancer than in benign disease or controls were observed [291]. These findings may indicate the potential role of tumor-derived sEVs (exosomes) in malignancy. sEVs (exosomes) released from the cancer cells have a strong capacity to affect cancer progression in several ways, including promotion of cancer cell migration [292], invasion [293], angiogenesis [234], vascular permeability [294], drug resistance [295], or intracellular communication during tumor development by autocrine and paracrine secretion of exosomal cargo (represented by proteins, metabolites, DNAs, RNAs, and miRNAs; Figure 2) [296]. Since miRNAs are considered as the major functional molecules of sEVs (exosomes) in intercellular communication, recruitment, and reprogramming important components of the tumor microenvironment [71], they are intensively studied among the exosomal RNA contents. Several studies indicated that sEVs (exosomes) contain high levels of miRNAs, which have been shown to contribute to metastasis [297], immunomodulation [298], chemoresistance [299], angiogenesis and vascular permeability [294] in multiple tumor types. Moreover, exosomal miRNAs were suggested as potential biomarkers for diagnosis and prognosis in various types of cancer, such as miR-320d for metastatic colorectal cancer [300], miR-10-5p for early-stage hepatocellular carcinoma [301], miR-106b for lung cancer [302] or miR-34a for ovarian cancer [303]. As sEVs’ (exosomes) isolation methods vary with respect to purity, a mixture of sEVs (exosomes) and other vesicles may be found in the isolated fraction. The identity of sEVs (exosomes) mentioned in this review was confirmed either by transmission electron microscopy [294,297,298,299,300,301,302,303] or by the presence of exosome-specific markers, such CD9, CD91, CD63, HSP70 or TSG101 [294,298,300,302,303], and size distribution (30–150 nm) was validated by nanoparticle tracking analysis [294,297,298,300].

sEVs (exosomes) research in veterinary medicine is still at an early stage, which is underlined by the first articles from 2017 [304,305,306,307]. The majority of recently published studies are mainly focused on canine and feline cancers [256,257,307,308,309,310,311,312,313,314]. Regarding exomiRs in canine cancer, our literature review revealed only a few studies analyzing miRNAs in canine oral melanoma [314], multicentric lymphomas [312], and lymphoid tumor cell lines [310].

To the best of our knowledge, in 2018, Fish et al. published the first and so far only study reporting the shedding of exosome-derived miRNA by canine mammary cells in vitro [309]. In particular, cell-free conditioned media containing exosome-like vesicles from three normal canine mammary epithelial cell cultures from canine patients without mammary pathology and five canine mammary tumor cell lines with histopathology-confirmed mammary carcinoma (CMT12, CMT27, CMT28, CMT47, CMT119) were used to yield a number of significantly upregulated and downregulated exomiRs that may represent putative biomarkers of mammary neoplasia. This complex study detected 338 unique exomiRs with 145 differentially expressed exomiRs (118 upregulated and 27 downregulated) having >±1.5-fold difference between tumor and normal samples.

Two proposed circulating low-invasive biomarkers in canine neoplastic diseases, including mammary carcinoma [315], miR-126, and miR-214, were also monitored in mammary tumors-exosomes. Generally, both miRNAs demonstrated a broad influence on cancer pathogenesis through the regulation of angiogenesis, proliferation, migration, and cancer cell death [316,317]. Therefore, alteration in their expression has a critical impact on tumor progression. In this study, miR-126 was found to be upregulated (2.25-fold). Thus, miR-126 may represent a prospective exosomal miRNA-based biomarker in canine mammary tumors. However, the expression of miR-214 was strongly downregulated (−9.13-fold) in the exosomal RNA of canine mammary tumors. As explained by the authors, high levels of miR-214 monitored in canine neoplastic diseases, including mammary cancer, can be either a result of secretion of other than canine mammary tumors cells (i.e., cells of the immune system, stroma or other organs) or a mismatch between tumor cell, exosomal and circulating miRNA profiles [309].

The findings of this study correlate with previously published studies on miRNAs in CMC discussed above. Several miRNAs, including miR-18a, miR-19a, miR-29b/c, miR-181a/b, miR-215, miR-345, miR-371, and miR-1841, were found to be upregulated in both canine mammary tumor cells and their exosomes [110,116,117,121]. However, some discrepancies in exomiRs expression levels compared to miRNAs profiles of tumor cells in other studies were observed, such as miR-19a, miR-29b/c, miR-31, miR-34c, miR-181a/b, miR-155, and miR-495 [113,114,117]. As discussed by the authors, this inconsistency may be a result of the active selection or enrichment process of particular miRNAs within exosomes or as a consequence of dramatic changes in tumor cell phenotype and gene expression in metastatic lesions [309].

In the same study, gene ontology enrichment analysis showed the cellular role of exomiRs in the regulation of enriched biological processes, such as positive regulation of cell proliferation, positive regulation of the apoptotic process, cell migration, response to hypoxia, regulation of gene expression, negative regulation of cell migration, or chromatin remodeling (histone ubiquitination or trimethylation) [309]. Target gene representation analysis associated with enriched gene ontology terms in order to select suitable candidates for clinical biomarker applications identified three miRNAs: miR-18a, miR-19a, and miR-181a [309]. These miRNAs were also the most significantly upregulated among all exomiRs (10.34-fold, 3.84-fold, and 7.70-fold, respectively) [309]. Moreover, miR-18a, miR-19a, and miR-181a were predicted in silico to target the estrogen receptor (ESR1α), the expression of which is known to be lost in human and canine neoplasms along with increasing grade and stage (miR-18a: miRDB target score = 99; miR-19a: miRDB target score = 71, and miR-181a: miRDB target score = 79) [309]. Based on these findings, the authors assume that miR-18a, miR-19a, and miR-181a represent non-invasive markers of hormone status and phenotype in CMC [309].

In contrast to CMC, there are several studies suggesting exosomal miRNAs as biomarkers for HBC. In these studies, several exomiRs were proposed as diagnostic biomarkers (miR-10b, miR-21, miR-101-3p, miR-105-5p, miR-134, miR-200c, miR-372) [219,318,319,320,321], prognostic biomarkers (miR-17-5p, miR-18a-5p, miR-20b-5p, miR-21, miR-29b-3p, miR-93-5p, miR-105, miR-124-3p, miR-130a-3p, miR-195-5p, miR-200c-3p, miR-338-3p, miR-340-5p, miR-486-5p, miR-1246) [219,319,322,323] of breast cancer, or indicators of a triple-negative phenotype (miR-373) [318].

Some of these HBC exomiRs were also identified in the canine study of Fish et al. [309]. In particular, cfa-miR-18a (hsa-mir-18a-5p), cfa-miR-20b (hsa-miR-20b-5p), cfa-miR-21 (hsa-miR-21), miR-29b (hsa-miR-29b-3p), miR-93 (hsa-miR-93-5p), cfa-miR-101 (hsa-miR-101-3p), cfa-miR-105a (hsa-miR-105-5p), cfa-miR-130a (hsa-miR-130a-3p), cfa-miR-200c (hsa-miR-200c-3p), cfa-miR-340 (hsa-340-5p), and cfa-miR-486 (hsa-miR-486-5p). These data show that CMC and HBC cells exclude sEVs (exosomes) enriched in differentially expressed miRNAs. The identification of exomiRs in exosomes excluded by cancer cells indicates the possibility of also detecting them in biofluids, such as blood or urine. This allows their use as non- or minimally invasive biomarkers. Furthermore, the observed similarity between CMC and HBC exomiRs profiles may have significance for translational research [309].

## 4. Conclusions

The non-exosomal and exosome-derived miRNAs identified in CMC as promising biomarkers reviewed in this study reveal heterogeneity in relation to expression level, potential use, and sampling (Table 2).

However, CMC mimics human breast tumors in many aspects (histopathology, clinical outcome or molecular markers). The similarities in terms of function and dynamics of miRNAs in mammary/breast cancer point to the role of these small non-coding RNAs in cancer mechanisms of both canine and human origin. MiRNAs are post-transcriptional regulators of gene expression with an impact on practically all cellular physiological and pathological processes. Since miRNAs are involved in cancer-related processes (such as carcinogenesis, cell proliferation, invasion, metastasis, apoptosis or chemoresistance), their diagnostic, prognostic, and therapeutic significance has been proposed. Over the past few years, several studies regarding miRNA-based biomarkers of mammary cancer have been carried out in canine patients. Most of these studies were focused on miRNAs derived from tumors or cancer cell lines. However, a traditional solid biopsy is gradually receding and more often is being replaced by liquid biopsy, wherein biofluid-extracted biomarkers provide a platform for non-invasive or minimally invasive diagnosis and prognosis. sEVs (exosomes) are present in many biological fluids and can be used similarly for minimally invasive liquid biopsies in veterinary medicine. Furthermore, their cargo plays an important role in various physiological and pathological processes. In particular, exosome-derived miRNAs have been shown to have a complex role in tumorigenesis and tumor progression. However, utilizing sEVs (exosomes) and their exomiRs cargo as a diagnostic tool for CMC is still in its infancy and requires further investigation. Moreover, most of the presented studies were conducted on small groups of patients. Although all of the above-mentioned miRNAs-based biomarkers seem to have diagnostic or prognostic potential in CMC, more detailed studies should be carried out in the near future.

## Figures and Tables

**Table 1:** Comparison of canine mammary cancer and human breast cancer.

Correlation Level	Similarities
clinical	onset age
	tumor incidence
	clinical stage
	metastasis
	lymph node invasion
	hormonal etiology
	disease course
pathological	long-term oncogenic environment
	intratumoral heterogeneity
	treatment resistance
molecular	up- or downregulation of adhesion molecules
	overexpression of growth factors
	low or high hormone expression
	upregulation of oncogenes
	downregulation of tumor suppressor genes
	elevated production of various proteins
	altered microRNA expression
etiological	sex hormones
	obesity

**Table 2:** Overview of non-exosomal and exosome-derived microRNAs (miRNAs) altered in canine mammary cancer with biomarker potential.

Non-Exosomal miRNAs				
miRNA	Expression Level	Potential Use	Sample	Reference
miR-21	up	diagnostic	tumor	[110,113,115]
	up	prognostic	blood serum	[119]
	up	metastatic	tumor	[114]
	up	diagnostic	blood serum	[120]
miR-29b	up	diagnostic	tumor	[110]
	up	diagnostic	blood serum	[121]
	up	diagnostic	cell line	[116]
	down	diagnostic	tumor	[113]
	down	prognostic	blood serum	[119]
	down	metastatic	tumor	[114]
miR-141	up	diagnostic and prognostic	cell line	[117]
miR-429	up	diagnostic	cell line	[117]
miR-200c	up	diagnostic	cell line	[117]
miR-497	down	therapeutic	cell line	[118]
miR-10b	down	metastatic	tumor	[113]
miR-101	down	metastatic	tumor	[113]
miR-125a/b	down	metastatic	tumor	[113,114]
miR-136	down	metastatic	tumor	[113]
miR-143	down	metastatic	tumor	[113]
	up	diagnostic	tumor	[114]
	up	diagnostic	cell line	[116]
miR-145	down	metastatic	tumor	[113]
let-7f	down	metastatic	tumor	[113]
miR-203	down	diagnostic	tumor	[113]
	up	metastatic	tumor	[114]
miR-210	up	diagnostic	tumor	[113,114]
miR-138a	down	diagnostic	cell line	[116]
miR-8832	down	diagnostic	tumor	[111]
miR-96	up	diagnostic	tumor	[111]
miR-149	down	diagnostic	tumor	[111]
miR-18a	up	prognostic	blood serum	[121]
miR-19b	up	diagnostic	blood serum	[121]
**Exosome-derived miRNAs**				
miR-126	up	diagnostic	conditioned medium	[309]
miR-214	down	diagnostic	conditioned medium	[309]
miR-18a	up	diagnostic	conditioned medium	[309]
miR-19a	up	diagnostic	conditioned medium	[309]
miR-181a	up	diagnostic	conditioned medium	[309]

Please note that miRNAs are listed based on their occurrence in the article.

## Data Availability

No new data were created or analyzed in this study. Data sharing is not applicable to this article.

## Abbreviations

*ADCY6*	gene encoding adenylyl cyclase type 6
Ago2	Argonaute 2
ALIX	apoptosis-linked gene 2-interacting protein X
BMI	body mass index
*BRCA1*	breast cancer gene 1
*BRCA2*	breast cancer gene 2
*BRPF3*	gene encoding bromodomain and PHD finger containing 3
CCT2	T-complex protein 1 subunit beta
*CDKN2A*	gene encoding cyclin-dependent kinase inhibitor 2A
CEA	carcinoembryonic antigen
cfa	*Canis lupus familiaris*
*c-KIT*	gene encoding tyrosine-protein kinase KIT
CMC	canine mammary cancer
DGCR8	DiGeorge syndrome critical region gene 8
dPCR	digital droplet PCR
EGF	epidermal growth factor
EGFR	epidermal growth factor receptor
EMT	epithelial-mesenchymal transition
ERRFI1	ERBB receptor feedback inhibitor 1
ERα	estrogen receptor alfa
ERCC1	DNA excision repair protein
ESCRT	endosomal sorting complexes required for transport
EVs	extracellular vesicles
*GNAO1*	gene encoding guanine nucleotide-binding protein-alpha O1
HBC	human breast cancer
HER2	human epidermal growth factor receptor 2
HER3	human epidermal growth factor receptor 3
HIF1α	hypoxia-inducible factor 1-alpha
hsa	*Homo sapiens*
HSP	heat shock protein
ICAM-1	intercellular adhesion molecule 1
IGF-1	insulin-like growth factor-1
ING3	human epidermal growth factor receptor 3
INK4	inhibitor of CDK4
*IRAK2*	gene encoding interleukin-1 receptor-associated kinase-like 2
ISEV	The International Society for Extracellular Vesicles
JNK	c-Jun N-terminal Kinase
*K-RAS*	gene for Kirsten rat sarcoma virus
*LRIG1*	gene encoding leucine-rich repeats and immunoglobulin-like domains 1
m/lEVs	medium/large extracellular vesicles
*MAPK*	gene encoding mitogen-activated protein kinase
miRNA	microRNA
MMP2	matrix metalloproteinase-2
mRNA	messenger RNA
MVB	multivesicular bodies
MYLIP	myosin regulatory light chain interacting protein
NF-ĸB	nuclear factor kappa-light-chain-enhancer of activated B cells
NGS	Nottingham histological grade
PACT	protein kinase RNA activator
PCNA	proliferating cell nuclear antigen
PCP	planar cell polarity
PCR	polymerase chain reaction
PECAM-1	platelet endothelial cell adhesion molecule-1
PI3K/AKT	phosphatidylinositol-4,5-bisphosphate 3-kinase/protein kinase B
Pre-miRNA	precursor miRNA
Pri-miRNA	primary miRNA
*PTEN*	gene encoding phosphatase and tensin homolog
RISC	RNA-induced silencing complex
*RNF2*	gene encoding E3 ubiquitin-protein ligase RING2
ROC-AUC	receiver operator characteristic-area under the curve
RT-PCR	real time-PCR
sEVs	small extracellular vesicles
SOX2	(sex determining region Y)-box 2
TNRC6A	trinucleotide repeat-containing gene 6A protein
*TP53*	gene encoding tumor protein 53
TRBP	transactivation response RNA binding protein
TSG101	tumor susceptibility gene 101 protein
*UGT1A1* UTR	gene encoding UDP-glucuronosyltransferase 1-1 enzyme untranslated regions
VEGF	vascular endothelial growth factor
VEGF-A	vascular endothelial growth factor A
VPS4	vacuolar protein sorting-associated protein
VTA1	vesicle trafficking 1
WHO	World Health Organisation
Wnt	Wingless and Int-1
YAP1	yes-associated protein 1
